# Childhood and current socioeconomic position as determinants of sedentary time among young and early midlife employees

**DOI:** 10.1093/eurpub/ckaf152

**Published:** 2025-09-01

**Authors:** Eero Kekäläinen, Ossi Rahkonen, Ville Päivärinne, Henriikka Nurminen, Tea Lallukka

**Affiliations:** Department of Public Health, University of Helsinki, Helsinki, Finland; Department of Public Health, University of Helsinki, Helsinki, Finland; Department of Public Health, University of Helsinki, Helsinki, Finland; Finnish Institute for Health and Welfare, Helsinki, Finland; Department of Public Health, University of Helsinki, Helsinki, Finland; Department of Public Health, University of Helsinki, Helsinki, Finland

## Abstract

Socioeconomic position (SEP) is one of the primary determinants of sedentary behaviour. The study investigated the associations between life-course socioeconomic circumstances and sedentary time (ST) among young and early midlife municipal employees. We used data from the 2017 Helsinki Health Study (*N* = 4532), which targeted 19- to 39-year-old employees of the City of Helsinki. SEP was assessed through both childhood and current indicators: parental and own educational level, childhood and current economic difficulties, occupational class, income, and wealth. ST was self-reported in minutes per weekday across five behavioural domains. Linear regression models examined differences in STs between socioeconomic groups, with 95% confidence intervals (CIs). All SEP indicators except childhood economic difficulties were associated with total ST. Participants in the highest income quartile reported 76 min (95% CI 60–92) more ST per day than those in the lowest quartile. Similarly, participants with higher education sat 69 min (95% CI 55–84) longer than those with lower education. The largest differences were observed during working hours, with higher education and income associated with more ST. In contrast, lower SEP was associated with more ST spent at home in front of a television/computer and in vehicles. Although individuals with higher SEP often engage in more physical activity and have better health behaviours overall, they are also the most sedentary, especially during work hours. The association between SEP and ST varies across behavioural domains, emphasizing the importance of context-specific interventions.

## Introduction

Sedentary behaviour (SB), from Latin *sedere*, meaning (‘to sit’), characterized by activities involving low energy expenditure, has become a growing public health concern, particularly given its associations with various adverse health outcomes [1–3]. Interruptions to sedentary time (ST) may mitigate these effects [4]. As our society becomes increasingly sedentary due to changes in work and lifestyle, such as increased screen time and greater automobile dependency, understanding the factors that contribute to such behaviour is becoming crucial.

Socioeconomic position (SEP), a multidimensional construct encompassing indicators such as education, occupation, and income [5], has been identified as a substantial, perhaps the most consistent, determinant of SB [6]. General population and children with lower SEP generally exhibit higher SB than those with higher SEP [7, 8]. Among adults, higher education is often associated with less screen-based and total ST, but more occupational and transport related sitting [6, 9]. Occupational class also affects ST; professional roles are more likely to involve high levels of occupational sitting than non-professional roles, while manual workers exhibit more SB outside of work [6]. Office work, despite being associated with higher levels of total ST, associates with less SB outside of work [6]. Higher income is typically associated with higher levels of overall ST, but less screen-based ST during leisure time [6]. Other socioeconomic indicators include housing tenure, wealth, and economic difficulties. It is also important to consider childhood socioeconomic circumstances, parental education level, and childhood economic difficulties, as adults from less advantaged childhood backgrounds tend to engage in physical activity less frequently [10]. However, most research on SEP and SB have primarily focused on adolescents [7, 8, 11, 12], older adults [8, 13], or the general population [6, 8, 9], and research on their associations among specific occupational cohorts is lacking. Previous studies have mainly focused on one or two SEP indicators, either childhood or current, thus overlooking the life-course perspective.

Considering this, the City of Helsinki offers a diverse workforce spanning from manual workers to professionals, providing an ideal setting to explore SEP–SB associations among young and early midlife employees. Given their age and occupational characteristics, this cohort helps address knowledge gaps, notably regarding younger adults with active career trajectories, who are often the focus of ST interventions [14–17].

In addition to SEP, sociodemographic and health-related factors are also linked to SB and should also be considered. In terms of demographic characteristics, studies suggest that older adults, men, single individuals, and unemployed people may be more sedentary than other groups [6, 9]. Alcohol consumption, low physical activity [6, 9], and insomnia [18] have been linked to higher levels of SB. In terms of health, chronic illnesses, depressive symptoms, anxiety, tension or stress, physical limitations, and a higher body mass index (BMI) have been associated with increased ST [6, 9]. However, the aforementioned factors can also be associated with SEP, and many are associated with both SEP and ST.

This study investigated how multiple life-course SEP measures relate to ST among young and early midlife municipal employees in Finland. The choice of socioeconomic indicators was based on previous studies of similar topics [19, 20]. ST is also examined across different domains, as previous studies have emphasized the importance of this [6, 21].

## Methods

### Study population

**Figure 1.**
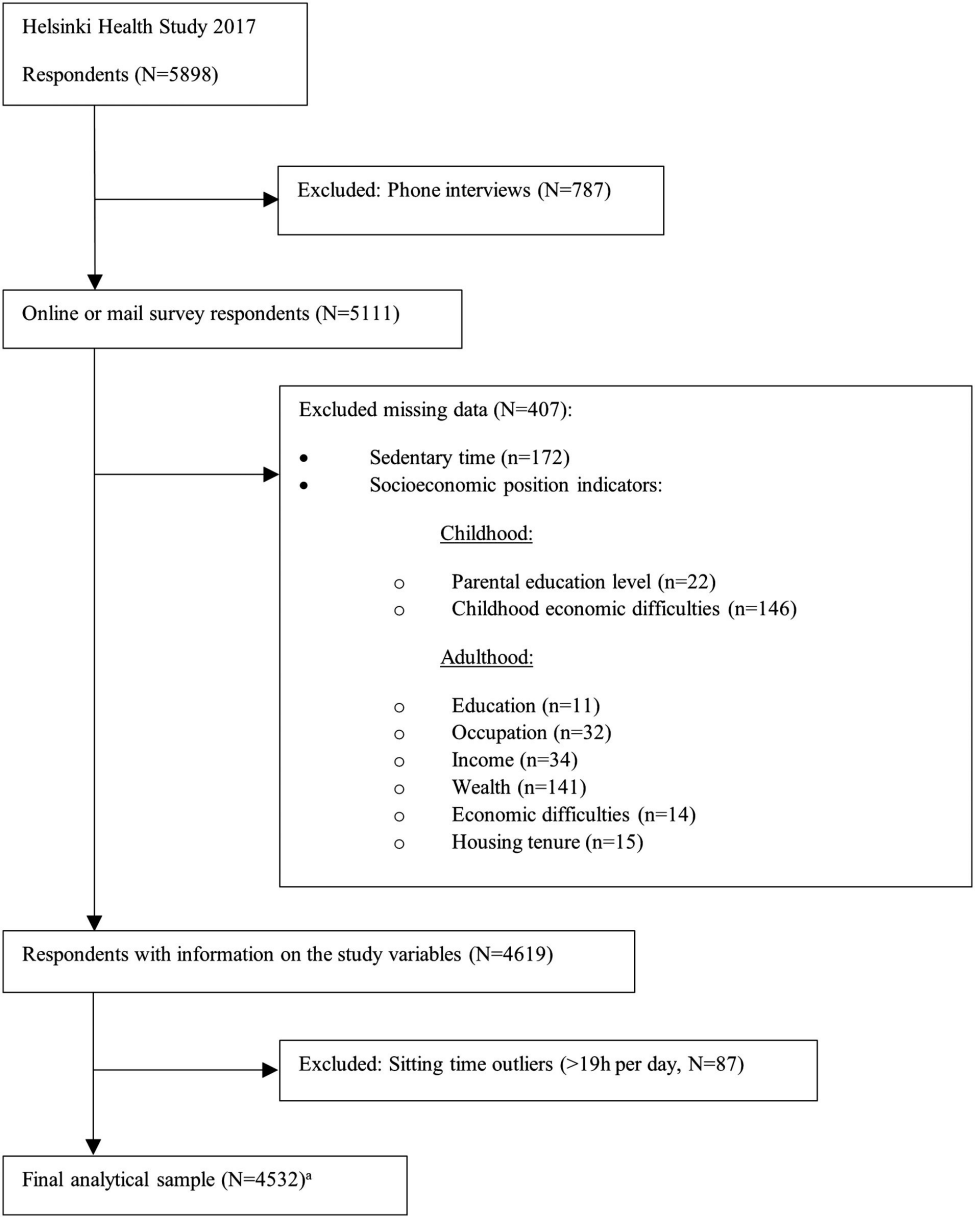
Flowchart of study participant selection. ^a^The sample size for the ‘During working hours’ domain was 4093, as participants who were not employed at the time of the survey were excluded.

Data came from the 2017 Helsinki Health Study (HHS) survey, targeting 19- to 39-year-old employees of the City of Helsinki (*N* = 5898, response rate 51.5%) [22]. Participants were recruited in autumn 2017 via workplaces and completed the survey online, by mail, or by phone for non-respondents to the full surveys. More detailed information on recruitment and procedures is available in the original HHS publication [22]. The phone respondents were excluded from the analysis because they were not asked the necessary questions about ST, as were the respondents who did not provide the required information on ST or socioeconomic indicators. The final sample included 4532 individuals (80% women). Exclusions are detailed in Fig. 1 and survey questions used in this study are provided in Supplementary File 1.

### Measuring socioeconomic circumstances

Seven indicators were used to measure childhood and current SEP. Childhood SEP was assessed using the highest education level of either parent (‘Elementary school’, ‘Vocational school’, ‘Upper secondary school’, ‘Higher education’) and by childhood economic difficulties, defined whether the family had experienced major economic difficulties before the participant turned 16.

Current SEP included participants’ education level, categorized as ‘Low’ (upper secondary school or lower), ‘Intermediate’ (bachelor’s degree), or ‘High’ (master’s degree or higher). Occupational class from register data was classified into four categories: ‘Manual worker’ (e.g. transport workers, cleaners), ‘Routine non-manual employee’ (e.g. clerical workers, care workers), ‘Semi-professional’ (e.g. nurses, technicians), and ‘Professional’ (e.g. teachers, doctors, managers). Household income was evaluated by estimating the combined disposable income of all the household members, adjusting it for household size according to the modified Organization for Economic Co-operation and Development (OECD) equivalence scale, and then divided into quartiles. Wealth was self-reported as total assets minus debts, categorized as ‘<10 000 €’, ‘10 000 €–99 999 €’ or ‘≥100 000 €’. Economic difficulties were assessed by frequency of trouble affording necessities or paying bills, categorized as ‘Frequent difficulties’, ‘Occasional difficulties’ or ‘No difficulties’.

The gender interaction tests on SEP indicators found considerable gender interactions in education and housing tenure (categorized as ‘Homeowners’ or ‘Renters/others’). Since the association between housing tenure and ST differed between men and women, with opposite directions of effect, combined linear regression results for housing tenure are not presented, as combining genders would have obscured these differences. Instead, we excluded housing tenure from the regression models to maintain consistency across analyses. For other SEP indicators, the genders were combined and handled as one sample, as the trends were similar across genders and the number of male respondents was low. In the case of education, the differences in ST followed a similar trend among both genders, enabling ‘one sample’ analysis.

### Measures of ST

The duration of SB was measured using a series of questions about the average time spent sitting on a weekday in five different domains: (1) time spent at home watching TV or using a computer, (2) at home reading, (3) in a vehicle, (4) during working hours, and (5) elsewhere. These domains align with those identified previously [23, 24].

ST data were slightly right-skewed, resulting in non-normally distributed residuals. To address this, outliers defined as participants with a total daily sitting time exceeding 19 hours were removed. The cut-off point was determined using the *z*-score method: a *z*-score of 3 was considered the threshold [25]. This process resulted in the removal of 87 participants from the dataset (Fig. 1).

### Covariates

Covariates included sociodemographic and health-related factors that have been associated with SB [6, 9, 18]. Following earlier procedures [20], missing values of the covariates (amounts ranging from 0% to 3.6%) were imputed into comparison groups, except for age and BMI, which were treated as continuous variables with missing values were replaced by the mean. BMI was calculated from self-reported height and weight.

The sociodemographic variables were gender, age, marital status (‘Married/cohabiting’ or ‘Other’), and work status (‘Working’ or ‘Not working’). Although all participants were employed by the City of Helsinki at the time of sampling, some were on temporary leave, and thus were labelled as ‘Not working’.

The health-related factors included leisure-time physical activity (LTPA), categorized into four groups according to metabolic equivalent (MET) hours: ‘Low activity’ (<20 MET-hours/week), ‘Moderate activity’ (>20 MET-hours/week of moderate intensity), ‘Vigorous activity’ (20–80 MET-hours/week, including vigorous activity), and ‘High vigorous activity’ (over 80 MET-hours/week, including vigorous activity) [26]. Sleep sufficiency was classified as ‘Sufficient’ (‘almost always’ or ‘often’) or ‘Insufficient’ (‘rarely or hardly ever’). Binge drinking was dichotomized based on a cut-off point of weekly or more frequently, resulting in two categories: ‘No binge drinking or missing’ and ‘Weekly or more frequently’. Self-rated health was dichotomized into ‘Good health’ (‘good’, ‘very good’, or ‘excellent’) and ‘Poor health’ (‘poor’ or ‘moderate’).

### Statistical analysis

Analysis of variance (ANOVA) was conducted to examine associations between study variables and ST.

Linear regression was used to examine the relationship between SEP indicators and total sitting time. Differences in sitting time between SEP categories were reported in minutes, along with their 95% confidence intervals. Model 1 was adjusted for gender and age. Model 2 consisted of Model 1, further adjusted for marital status and work status. Model 3 consisted of Model 1, further adjusted for LTPA, sleep adequacy, binge drinking, BMI, and health. Model 4 was a fully adjusted model including all the covariates. There was a strong correlation (Spearman’s correlation) between occupational class and education (*r* = 0.783), and housing tenure and wealth (*r* = 0.590), as seen in Supplementary Table S1. To avoid multicollinearity, regression models were not simultaneously adjusted for SEP indicators.

Linear regression analyses were also conducted in five different domains separately, while adjusted for age and gender. If a participant had not provided sitting time in one domain but had provided it in other domains, the sitting time for the missing domain was set to zero. The same sample size (*n* = 4532) was used for all the domains except work, from which the participants who were not employed at the time were excluded (*n* = 4093).

All the analyses were conducted using R version 4.4.0.

## Results

### Descriptive results

Of the participants, 90% were employed either full or part time, and men were more likely to be employed than women (97% vs 89%, *P* < .001). Table 1 presents the data characteristics of categorical variables. Men more often had low education (42% vs 32%) and manual jobs (14% vs 3%), while women were more often semi-professionals (43% vs 30%), but had more often wealth less than 10 000 € (36% vs 29%) (all *P* < .001).

**Table 1.** Data characteristics^a^

Variable	Category	Overall	Women	Men
*N*		4532	3615	917
Age (years)	<30	1448 (32.0)	1192 (33.0)	256 (27.9)
	≥30	3084 (68.0)	2423 (67.0)	661 (72.1)
Marital status	Married or cohabiting	3024 (66.7)	2364 (65.4)	660 (72.0)
	Other	1508 (33.3)	1251 (34.6)	257 (28.0)
Work status	Not working	439 (9.7)	412 (11.4)	27 (2.9)
	Working	4093 (90.3)	3203 (88.6)	890 (97.1)
Parental education level	Elementary school	414 (9.1)	325 (9.0)	89 (9.7)
	Vocational school	1586 (35.0)	1301 (36.0)	285 (31.1)
	Upper secondary school	570 (12.6)	451 (12.5)	119 (13.0)
	Higher education	1962 (43.3)	1538 (42.5)	424 (46.2)
Childhood economic difficulties	Yes	980 (21.6)	763 (21.1)	217 (23.7)
	No	3552 (78.4)	2852 (78.9)	700 (76.3)
Education level	Low	1522 (33.6)	1140 (31.5)	382 (41.7)
	Intermediate	1659 (36.6)	1390 (38.5)	269 (29.3)
	High	1351 (29.8)	1085 (30.0)	266 (29.0)
Occupational class	Manual worker	235 (5.2)	105 (2.9)	130 (14.2)
	Routine non-manual employee	1221 (26.9)	994 (27.5)	227 (24.8)
	Semi-professional	1812 (40.0)	1541 (42.6)	271 (29.6)
	Professional	1264 (27.9)	975 (27.0)	289 (31.5)
Income quartile	1 (lowest)	1134 (25.0)	904 (25.0)	230 (25.1)
	2	1138 (25.1)	924 (25.6)	214 (23.3)
	3	1126 (24.8)	883 (24.4)	243 (26.5)
	4 (highest)	1134 (25.0)	904 (25.0)	230 (25.1)
Wealth	<10 000 €	1580 (34.9)	1314 (36.3)	266 (29.0)
	10 000 €–99 999 €	1830 (40.4)	1406 (38.9)	424 (46.2)
	≥100 000 €	1122 (24.8)	895 (24.8)	227 (24.8)
Economic difficulties	Frequent difficulties	217 (4.8)	168 (4.6)	49 (5.3)
	Occasional difficulties	1271 (28.0)	1032 (28.5)	239 (26.1)
	No difficulties	3044 (67.2)	2415 (66.8)	629 (68.6)
Housing tenure	Renting/other	2583 (57.0)	2071 (57.3)	512 (55.8)
	Homeowner	1949 (43.0)	1544 (42.7)	405 (44.2)
Leisure-time physical activity	High vigorous activity	1016 (22.4)	743 (20.6)	273 (29.8)
	Vigorous activity	1709 (37.7)	1350 (37.3)	359 (39.1)
	Moderate activity	1111 (24.5)	958 (26.5)	153 (16.7)
	Low activity	696 (15.4)	564 (15.6)	132 (14.4)
Sleep sufficiency	Sufficient	3060 (67.5)	2425 (67.1)	635 (69.2)
	Insufficient	1472 (32.5)	1190 (32.9)	282 (30.8)
Binge drinking	No binge drinking or missing	4253 (93.8)	3480 (96.3)	773 (84.3)
	Weekly or more frequently	279 (6.2)	135 (3.7)	144 (15.7)
Body mass index (kg/m^2^)	<30	3889 (85.8)	3100 (85.8)	789 (86.0)
	≥30	643 (14.2)	515 (14.2)	128 (14.0)
Self-reported health	Good health	4058 (89.5)	3236 (89.5)	822 (89.6)
	Poor health	474 (10.5)	379 (10.5)	95 (10.4)
Total sedentary time	Low (<5.5 h)	1514 (33.4)	1284 (35.5)	230 (25.1)
	Intermediate (5.5–8.5 h)	1554 (34.3)	1247 (34.5)	307 (33.5)
	High (>8.5 h)	1464 (32.3)	1084 (30.0)	380 (41.4)

a The table presents the 2017 Helsinki Health Study variables stratified by gender and shows the amounts with percentages in brackets. It also presents the *P*-values to show the difference between genders.

The average daily ST was 7 h and 13 min, with an average of 3 h and 18 min spent sedentary during work. Men, on average, sat for longer than women (*P* < .001), with men sitting for 8 h and women for 7 h. Still, a higher proportion of men had high vigorous activity (29.8% vs 20.6%, *P* < .001). The data characteristics for the continuous variables are present in Supplementary Table S2.

Gender and education showed the strongest ST associations (*P* < .001), as indicated by ANOVA-results present in Supplementary Table S3. Other variables with significant associations included marital status, work status, parental education, occupational class, income, LTPA, sleep sufficiency, binge drinking, BMI, and self-reported health (all *P* < .05). In contrast, age (*P* = .208), childhood economic difficulties (*P* = .525), wealth (*P* = .635), current economic difficulties (*P* = .365), and housing tenure (*P* = .544) were not significantly associated with ST.

### SEP inequalities in ST

**Figure 2.**
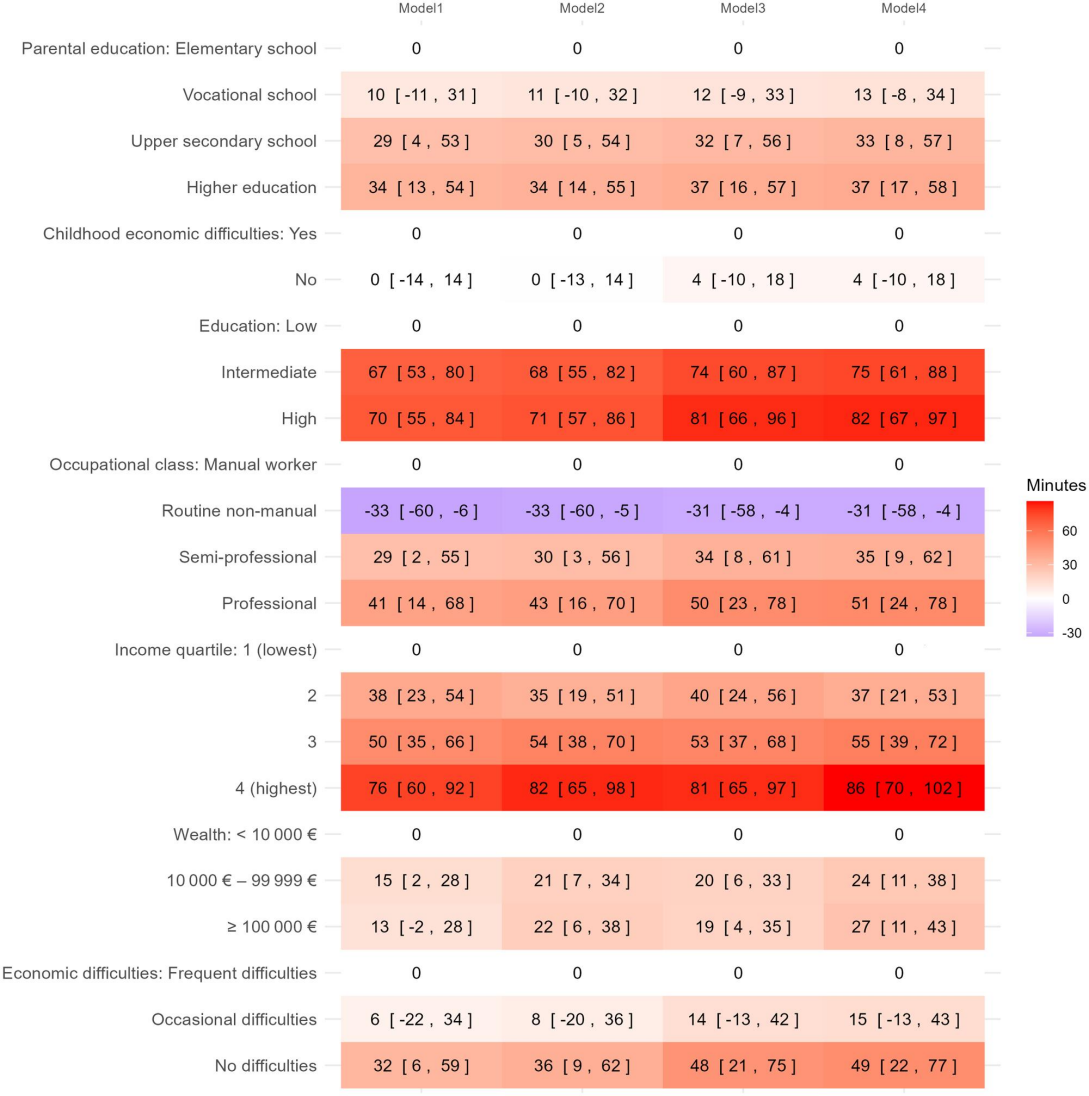
Linear regression analyses between indicators of socioeconomic position (SEP) and total sedentary time (differences in minutes), adjusted for different variables in different models,^a^ based on the 2017 Helsinki Health Study (*n* = 4532). ^a^Model 1 (adjusted for age, gender), Model 2 (Model 1 + marital status and work status), Model 3 (Model 1 + leisure-time physical activity, sleep adequacy, binge drinking, body mass index, and health), and Model 4 (fully adjusted for all covariates). The reference category for each of the seven SEP indicators is the lowest category, which is shown in the first row. The results are presented as differences in minutes, along with the 95% confidence intervals (CIs) in square brackets.

Linear regression analyses between SEP indicators and total ST showed that six of seven SEP indicators (parental and own education, occupational class, income, wealth, and current economic difficulties) were associated to total ST. Results are present in Fig. 2. The largest differences in total ST on a typical weekday were between the highest and the lowest income groups (76 min, 95% CI 60–92), professionals and routine non-manual employees (74 min, 95% CI 59–90), and participants with high versus low levels of education (70 min, 95% CI 55–84). Routine non-manual employees were less sedentary than manual workers (−33 min, 95% CI −60 to −6).

Adjusting for covariates only made a small contribution to the association, with minor increases observed in all SEP indicators. The largest increase occurred in the fully adjusted model, in which the difference in ST between income groups increased from 76 min (95% CI 60–92) to 86 min (95% CI 70–102).

### ST in different domains

**Figure 3.**
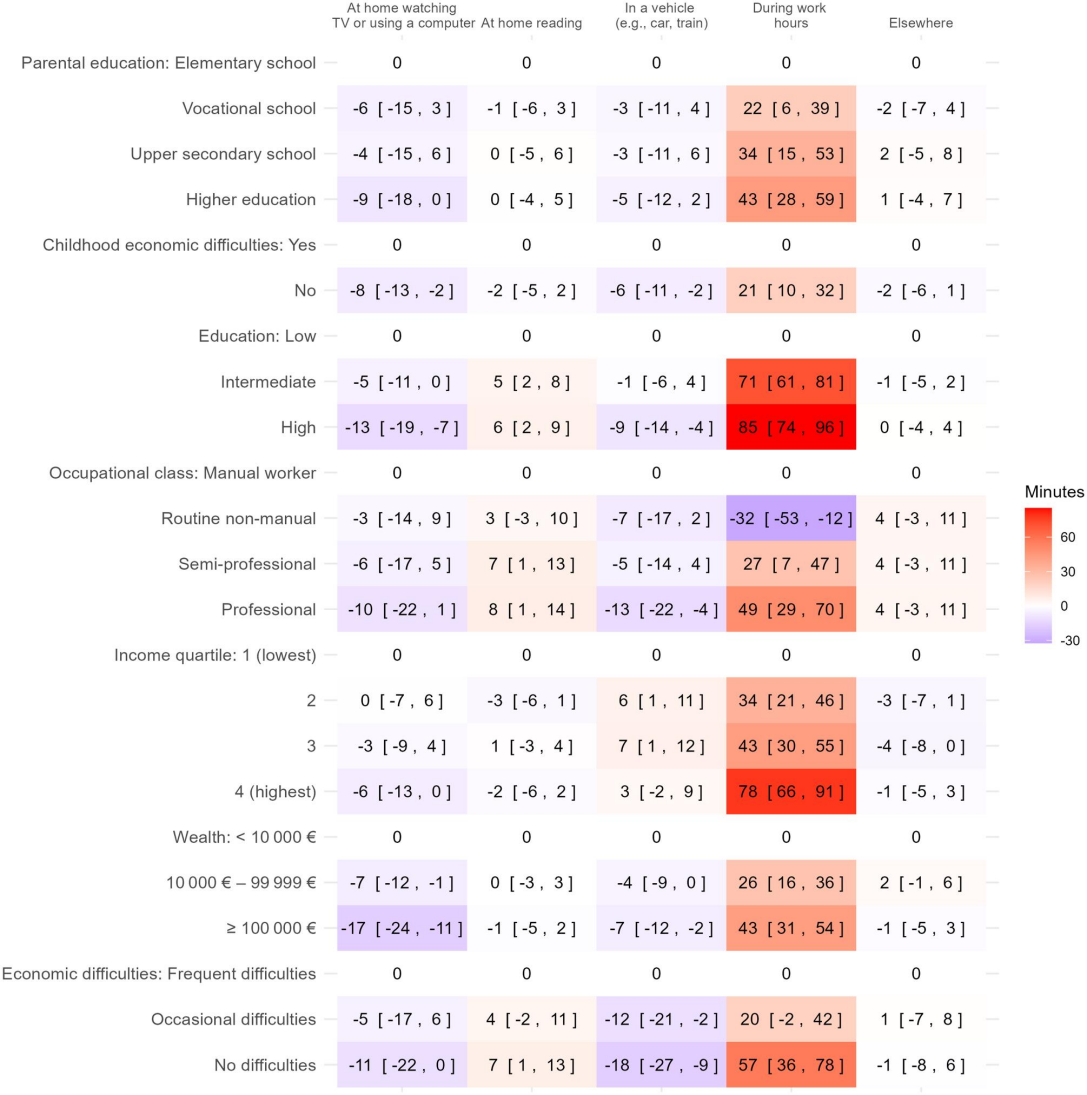
Linear regression analyses between indicators of socioeconomic position (SEP) and sedentary time across different domains (differences in minutes),^a^ based on the 2017 Helsinki Health Study (*n* = 4532). Sample size of ‘During working hours’ domain = 4093. ^a^Each of the seven SEP indicators’ categories are compared to the lowest category of the specified SEP, which is shown in the first row. The results are presented as a difference in minutes, along with their 95% confidence intervals (CIs) in square brackets. All domains were adjusted for age and gender.

Figure 3 presents the results of the linear regression analyses between SEP indicators and ST across different domains. The greatest differences in ST among all the SEP indicators were observed in the ‘During working hours’ domain. Like total ST, sitting during work hours was most strongly associated with higher education levels, professional occupational class, and higher income levels. However, unlike total ST, all the seven SEP indicators showed differences in ST during working hours. In addition, greater ST while reading was only weakly associated with the absence of economic difficulties, higher education level, and higher occupational class.

However, higher SEP was mainly associated with less ST in other domains. Greater wealth, higher education level, having no childhood or current economic difficulties, and higher parental education level were associated with less ST at home watching TV or using a computer. Similarly, having no economic difficulties, greater wealth, higher education level, and being in a professional occupational class were associated with less ST in a vehicle.

## Discussion

### Main findings

The most evident contributors to total SB were income, occupational class, and education. Of childhood socioeconomic circumstances, higher parental education level was associated with longer total ST. These associations remained largely unchanged after adjusting for sociodemographic and health factors.

All SEP indicators were associated with ST during working hours. This can partly be explained by most of the sitting occurring at work, and lower SEP might be linked to more physically demanding jobs. The observation that routine non-manual employees are less sedentary than manual workers, despite being in a higher SEP position, can be attributed to their work environment in this cohort. Care workers (routine non-manual employees) work in environments that encourage movement, and healthcare settings require constant mobility. In contrast, transport workers (manual workers) have tasks that involve prolonged periods of sitting or being in a vehicle. Professionals (e.g. teachers, doctors, managers) remain the most sedentary, as the nature of their work involves extensive desk tasks, meetings, and computer use.

Longer ST while reading at home was associated with having no economic difficulties, higher education level, and higher professional occupational class, though these associations were weak. This may reflect greater access to cognitively stimulating leisure activities among individuals with higher SEP, and that they also strive to engage in such activities [27].

In contrast, higher SEP (greater wealth, higher education level, and having no childhood or current economic difficulties) was associated with slightly less ST at home in front of the TV or computer. Similarly, having no economic difficulties, greater wealth, and higher education level were associated with slightly shorter ST in vehicles. This may be explained by greater access to alternative leisure options among higher SEP individuals, such as recreational facilities, or outdoor environments [27]. Wealthier individuals may also live in neighbourhoods with better walkability, more green spaces, and safer environments, which can reduce reliance on screen-based or passive leisure [28].

Conversely, individuals with lower SEP may experience greater occupational fatigue due to physically demanding jobs, which could increase the appeal of sedentary leisure activities like TV watching [29]. Limited access to safe or affordable recreational spaces may further constrain opportunities for active leisure [30].

### Previous studies

Previous research has found that lower SEP is associated with higher levels of SB in the general population and among children [7, 8], a finding that is in contrast with the results for municipal employees in this study. This difference is best explained by the composition of our cohort, which consisted entirely of employed individuals, for whom workplace sitting dominated overall ST.

Prior studies have also demonstrated that higher education among adults is generally linked to less TV watching and screen-based ST, but more occupational sitting and sitting during transportation [6, 9]. Although our results align with these findings regarding TV watching and occupational ST, we found that individuals with a higher education level and occupational class also exhibited more total ST and more ST at home reading. Research has shown that manual work among adults correlates positively with SB outside of work, whereas office work is linked to higher total ST, despite being associated with less SB during non-working hours [6]. This is in line with our results, according to which professional classes showed more workplace sitting but potentially less sitting outside of work.

Income has shown to be associated with overall ST but to negatively correlate with screen-based SB during leisure-time among adults [6]. Although we observed a similar trend, the association between income and screen-based ST was not statistically significant in our study. Overall wealth was associated with less screen-based ST, as previously observed in a study of preschool children [12]. This may be because higher income often enables individuals to access a wider range of leisure activities that do not involve screens, such as reading, hobbies, or participation in clubs and sports.

To our knowledge, no studies have examined the association between childhood SEP and adulthood ST, though the association with childhood ST has been widely studied. Our results regarding the association between higher parental education level and less screen-based ST mirrors those found during childhood [11]. However, we found that longer ST during working hours is associated with higher parental education level. After adjusting total ST to the individual’s own education, the difference between ST among the parental education categories ‘Higher education level’ and ‘Elementary school’ decreased from 34 to 9 min. Therefore, the effect of parental education is mostly explained by the individual’s own education, as this is strongly influenced by parental education.

### Implications for policy and interventions

This study indicates that, in contrast to general health trends, employees with high SEP are most sedentary, especially during their working hours. This highlights the need for interventions that consider the context and socioeconomic background of individuals.

For higher SEP groups, particularly professionals in sedentary occupations, workplace strategies, like promoting the use of standing desks, walking meetings, scheduled movement breaks, and ergonomic office design, should be prioritized. Employers can foster a culture that encourages physical activity during the workday.

For lower SEP groups, who may face barriers to active leisure due to fatigue or limited access to recreational facilities, community-level strategies are suggested. These could involve improving access to affordable or free physical activity programs, enhancing neighbourhood walkability, and investing in safe public spaces and green areas.

Public health policies should adopt a multi-level approach that addresses both occupational and leisure-time SB, ensuring that interventions are equitable and responsive to the needs of diverse socioeconomic groups.

### Methodological considerations

All the participants were 19–39 years old municipal employees, 80% of whom were women, limiting the generalizability to other employment sectors, men, and people of other ages. Excluding phone respondents, who had somewhat lower SEPs, and non-respondents, who were more likely men, manual workers, and lower-income individuals, may have introduced selection bias. However, we found that the data mostly represented the target population [22].

Self-reported data may involve response bias, as participants may underreport ST or their criteria for what constitutes sitting may vary. Although ST questionnaires have not been formally validated, epidemiological studies commonly employ similar measures [21, 23]. According to several meta-analyses, sensor-based measurements are more reliable than self-reported ST [31]. However, sensor-based measurements do not observe the place in which ST occurs, and questionnaires are easier to carry out among large study groups compared to sensor-based measurements.

A cross-sectional design limits causal inference, especially if the associations are reciprocal. However, socioeconomic indicators tend to be relatively stable characteristics, and SB is unlikely to have a relevant, direct impact on changes in SEP and certainly has no effect on childhood SEP.

It is important to note that in this study, ‘SB’ was defined solely based on questions related to sitting, even though lying down and standing also have low MET values. We used the term ‘SB’ because previous studies that have used surveys based on sitting time have also used this term [6, 13, 21].

This study has also several notable strengths. It utilized a broad set of SEP indicators, encompassing both childhood and current SEP, enabling a life-course perspective on the determinants of SB. In addition to conventional indicators, we also considered the broader material aspects of SEP. Relevant sociodemographic and health-related covariates were included in the analyses, enhancing the robustness of the findings. Multiple SB domains were analysed alongside total ST, aligning with recent research recommendations [21], and reporting ST differences in minutes enhances interpretability.

Moreover, the study was based on a large dataset of employees from Finland’s largest employer, increasing statistical power and the robustness of results in the context of large-scale occupational research.

## Conclusions

Our findings show that, contrary to general health patterns, municipal employees with higher SEP are the most sedentary, particularly during their working hours. The association between SEP and ST varies across behavioural domains, emphasizing the importance of context-specific interventions. Among higher SEP groups, interventions should prioritize reducing occupational sitting, while among lower SEP groups, targeting leisure-time sitting may be more effective. This highlights the need for public health strategies that address SB across all socioeconomic groups. Tackling these disparities is essential for promoting health and well-being among individuals, including the working population.

## Supplementary Material

ckaf152_Supplementary_Data

## Data Availability

The Helsinki Health Study survey data (and the register data of the City of Helsinki) cannot be made publicly available due to strict data protection laws and regulations. The data can only be used for scientific research. More information on the survey data can be requested from the Helsinki Health Study research group (kttl-hhs@helsinki.fi).
