# Supplementary material for: Childhood and current socioeconomic position as determinants of sedentary time among young and early midlife employees
Source: Eur J Public Health. 2025 Sep 1;35(5):916–24. doi: 10.1093/eurpub/ckaf152 (PMC12529275; doi:10.1093/eurpub/ckaf152)
Supplement: ckaf152_Supplementary_Data [file ckaf152_supplementary_data.zip › ckaf152_Supplementary_Data/ejph-2025-01-om-0032-File002.docx]

S1 File. Additional methods Supplement: Survey questions used in the study

*Note: Most participants responded in Finnish or Swedish. The following is an English translation of the survey questions, which was also provided as an optional language version in the questionnaire.*

BACKGROUND INFORMATION

1. Are you a
   - man
   - woman
2. What is your year of birth? ________
3. What is the highest level of examination or qualification that you have attained?
   - elementary school, primary school, lower secondary school, middle school or less
   - vocational qualification
   - upper secondary school / matriculation / A-levels
   - Bachelor’s degree (for example UAS or university)
   - Master’s degree (for example UAS or university)
   - doctoral degree
4. What is/was your mother's highest level of education?
   - elementary school, primary school, middle school or less
   - vocational school, college or equivalent
   - upper secondary school / matriculation / A-levels
   - higher education degree/qualification / university degree
5. What is/was your father's highest level of education?
   - elementary school, primary school, middle school or less
   - vocational school, college or equivalent
   - upper secondary school / matriculation / A-levels
   - higher education degree/qualification / university degree
6. Are you currently mainly:
   - in full-time work
   - in part-time work
   - on family leave (parental leave, maternity leave, paternity leave etc.)
   - a student
   - long-term (over 6 months) sick leave
   - a recipient of rehabilitation allowance
   - on disability pension
   - unemployed
   - other
7. What is your marital status?
   - single (never married)
   - cohabiting
   - married or in a registered partnership
   - separated or divorced
   - widowed
8. What type of property do you live in?
   - owner occupier flat/house
   - right-of-occupancy or part-ownership flat/house
   - flat owned by your employer (City of Helsinki)
   - private rented property or a council flat
   - other type of housing
9. Does anyone live in your household besides you? You can choose several options.
   - no
   - spouse or partner
   - any other adults (spouse/partner excluded), how many? ________
   - children aged 0–18, how many? ________
10. In which of the following income categories does your household belong? Estimate the combined income of all members of your household, minus taxes and including any housing benefit, child benefit and any other benefits, allowances or transfers of income, in a typical month.
    - less than 1300 euro
    - 1300–1599 euro
    - 1600–1999 euro
    - 2000–2599 euro
    - 2600–3199 euro
    - 3200–3899 euro
    - 3900–4499 euro
    - 4500–5399 euro
    - 5400–6400 euro
    - more than 6400 euro
11. Did your family have major financial difficulties during your childhood (that is, up until you were 16)?
    - Yes
    - No

ALCOHOL

1. The next question concerns situations in which you drink six or more servings of alcoholic beverages at one sitting. Six or more servings is equivalent to at least:

- 4 pints (0.5 l each) medium-strength beer/mild cider or

- 3 pints (0.5 l each) strong beer/strong cider or

- one bottle (0.75 l) of mild wine (12%) or

- 6 restaurant servings (4 cl each) of spirits

How often do you drink six or more servings of alcoholic beverages at one sitting?

- - never
  - less than once a month
  - once a month
  - once a week
  - a few times a week
  - every day or almost every day

EXERCISE

1. Next, we will be asking about physical activity during your leisure and commuting time over the past 12 months. We have divided physical activities in four levels of exertion. First, estimate the exertion level of the physical activities you are engaged in. Then, estimate how often you engage in a physical activity equivalent to each level of exertion during one week rounded to closest 15 minutes (e.g. 02 hours and 45 minutes).
   1. During your leisure time

| **Strenuousness of**  **exercise** | **Hours** | **Minutes** |
| --- | --- | --- |
| Equivalent to walking | ________ | ________ |
| Equivalent to brisk walking | ________ | ________ |
| Equivalent to light running (jogging) | ________ | ________ |
| Equivalent to brisk running | ________ | ________ |

- 1. During your commute

| **Strenuousness of**  **exercise** | **Hours** | **Minutes** |
| --- | --- | --- |
| Equivalent to walking | ________ | ________ |
| Equivalent to brisk walking | ________ | ________ |
| Equivalent to light running (jogging) | ________ | ________ |
| Equivalent to brisk running | ________ | ________ |

1. How much time on average do you sit each weekday rounded to closest 15 minutes? Enter 0, if you spend no time sitting at all.

|  | **Hours** | **Minutes** |
| --- | --- | --- |
| At home watching television or sitting in front of the computer | ________ | ________ |
| At home reading | ________ | ________ |
| Sitting in a vehicle (e.g. car, train) | ________ | ________ |
| At work | ________ | ________ |
| Elsewhere | ________ | ________ |

HEIGHT

1. How tall are you? ________cm
2. How much do you weigh? ________kg (round to the nearest kilogram)

SLEEP

1. Do you feel that you get enough sleep?
   - yes, almost always
   - yes, often
   - rarely or hardly ever
2. In general, would you say your health is:
   - excellent
   - very good
   - good
   - fair
   - poor

FINANCIAL ASSETS AND INCOME

1. How often do you have enough money to afford the kind of food or clothing you/your family should have?
   - always
   - often
   - sometimes
   - seldom
   - never
2. How much difficulty do you have in meeting the payment of bills?
   - very little or none
   - slight
   - some
   - great
   - very great
3. If you sold all the assets in your own household, for example, your house, car, summer house and boat, cashed in your savings and investments, and paid off all your debts (including your mortgage), how much money do you think your household would have?
   - less than 10 000 euro
   - 10 000 – 49 999 euro
   - 50 000–99 999 euro
   - 100 000–199 999 euro
   - 200 000–299 999 euro
   - 300 000–399 999 euro
   - 400 000–499 999 euro
   - 500 000–699 999 euro
   - 700 000–900 000 euro
   - more than 900 000 euro
