# Supplementary material for: Childhood and current socioeconomic position as determinants of sedentary time among young and early midlife employees
Source: Eur J Public Health. 2025 Sep 1;35(5):916–24. doi: 10.1093/eurpub/ckaf152 (PMC12529275; doi:10.1093/eurpub/ckaf152)
Supplement: ckaf152_Supplementary_Data [file ckaf152_supplementary_data.zip › ckaf152_Supplementary_Data/ejph-2025-01-om-0032-File003.docx]

*Supplementary Table 1. Spearman’s correlation analysis results and gender-specific generalised variance inflation factors (GVIF) for the eight socioeconomic position indicators, based on the 2017 Helsinki Health Study (n=4532).*

|  | Women GVIF | Parental education level | Childhood economic difficulties | Education | Occupational class | Income | Wealth | Economic difficulties categories | Housing tenure | Men GVIF |
| --- | --- | --- | --- | --- | --- | --- | --- | --- | --- | --- |
| Parental education level | 1.120 | 1 | 0.153 | 0.266 | 0.245 | 0.133 | 0.103 | 0.090 | 0.056 | 1.164 |
| Childhood economic difficulties | 1.057 | 0.153 | 1 | 0.113 | 0.094 | 0.091 | 0.155 | 0.169 | 0.078 | 1.138 |
| Education level | 5.567 | 0.266 | 0.113 | 1 | 0.784 | 0.357 | 0.268 | 0.243 | 0.211 | 3.050 |
| Occupational class | 5.347 | 0.245 | 0.094 | 0.784 | 1 | 0.342 | 0.209 | 0.216 | 0.172 | 3.073 |
| Income | 1.580 | 0.133 | 0.091 | 0.357 | 0.342 | 1 | 0.279 | 0.270 | 0.213 | 1.533 |
| Wealth | 1.280 | 0.103 | 0.155 | 0.268 | 0.209 | 0.279 | 1 | 0.298 | 0.590 | 1.315 |
| Economic difficulties categories | 1.784 | 0.090 | 0.169 | 0.243 | 0.216 | 0.270 | 0.298 | 1 | 0.151 | 1.767 |
| Housing tenure | 1.210 | 0.056 | 0.078 | 0.211 | 0.172 | 0.213 | 0.590 | 0.151 | 1 | 1.287 |
