# Supplementary material for: Childhood and current socioeconomic position as determinants of sedentary time among young and early midlife employees
Source: Eur J Public Health. 2025 Sep 1;35(5):916–24. doi: 10.1093/eurpub/ckaf152 (PMC12529275; doi:10.1093/eurpub/ckaf152)
Supplement: ckaf152_Supplementary_Data [file ckaf152_supplementary_data.zip › ckaf152_Supplementary_Data/ejph-2025-01-om-0032-File005.docx]

*Supplementary Table 2. Data characteristics for continuous variables represented in means and standard deviations in brackets, based on the 2017 Helsinki Health Study (n=4532). P-values show the difference between genders.*

|  | Overall | Women | Men | p-value |
| --- | --- | --- | --- | --- |
| n | 4532 | 3615 | 917 |  |
| Total sedentary time | 7h 13min (3h 15min) | 7h 1min (3h 10min) | 8h 2min (3h 26min) | <0.001 |
| Sedentary time in minutes: |  |  |  |  |
| Total | 433 (195) | 421 (190) | 482 (206) | <0.001 |
| At home watching TV or using a computer | 124 (82) | 119 (79) | 145 (91) | <0.001 |
| At home reading | 40 (45) | 41 (45) | 39 (44) | 0.337 |
| In a vehicle (e.g. car, train) | 61 (67) | 60 (66) | 64.82 (70) | 0.059 |
| During working hours | 198 (142) | 192 (141) | 221 (144) | <0.001 |
| Elsewhere | 32 (56) | 31 (57) | 33 (52) | 0.488 |
| Age (in years) | 31.94 (4.59) | 31.82 (4.6) | 32.40 (4.49) | 0.001 |
| Body mass index (kg/m^2^) | 25.22 (5.15) | 25.03 (5.36) | 25.99 (4.15) | <0.001 |
