# Supplementary material for: Childhood and current socioeconomic position as determinants of sedentary time among young and early midlife employees
Source: Eur J Public Health. 2025 Sep 1;35(5):916–24. doi: 10.1093/eurpub/ckaf152 (PMC12529275; doi:10.1093/eurpub/ckaf152)
Supplement: ckaf152_Supplementary_Data [file ckaf152_supplementary_data.zip › ckaf152_Supplementary_Data/ejph-2025-01-om-0032-File006.docx]

*Supplementary Table 3. Mean sedentary time minutes with 95% confidence intervals for each category of the study variables and Analysis of Variance (ANOVA) results, based on the 2017 Helsinki Health Study (n=4532).*

| **Variable** | **Category** | **Mean**  **sedentary**  **time (min)** | **Confidence**  **intervals (min)** | **ANOVA**  **F-value** | **ANOVA**  **p-value** |
| --- | --- | --- | --- | --- | --- |
| Gender | Women | 421 | 414–427 | 78.507 | <0.001 |
|  | Men | 482 | 469–496 |  |  |
| Age (years) | <30 | 437 | 426–447 | 1.586 | 0.208 |
|  | ≥30 | 431 | 425–438 |  |  |
| Marital status | Married or cohabiting | 427 | 420–434 | 10.634 | 0.001 |
|  | Other | 444 | 435–454 |  |  |
| Work status | Not working | 401 | 383–419 | 5.842 | 0.016 |
|  | Working | 437 | 431–442 |  |  |
| Parental education level | Elementary school | 412 | 393–431 | 6.578 | <0.001 |
|  | Vocational school | 420 | 411–430 |  |  |
|  | Upper secondary school | 440 | 425–456 |  |  |
|  | Higher education | 446 | 437–454 |  |  |
| Childhood economic difficulties | Yes | 434 | 421–447 | 0.404 | 0.525 |
|  | No | 433 | 427–439 |  |  |
| Education level | Low | 394 | 384–404 | 58.265 | <0.001 |
|  | Intermediate | 452 | 443–461 |  |  |
|  | High | 454 | 444–463 |  |  |
| Occupational class | Manual worker | 442 | 415–470 | 5.333 | 0.001 |
|  | Routine non-manual employee | 386 | 375–398 |  |  |
|  | Semi-professional | 445 | 436–454 |  |  |
|  | Professional | 460 | 450–470 |  |  |
| Income quartile | 1 (lowest) | 392 | 381–403 | 16.450 | <0.001 |
|  | 2 | 429 | 418–441 |  |  |
|  | 3 | 443 | 432–455 |  |  |
|  | 4 (highest) | 468 | 457–478 |  |  |
| Wealth | <10 000 € | 423 | 413–433 | 0.455 | 0.635 |
|  | 10 000 € – 99 999 € | 441 | 432–450 |  |  |
|  | ≥100 000 € | 435 | 425–446 |  |  |
| Economic difficulties | Frequent difficulties | 411 | 385–437 | 1.008 | 0.365 |
|  | Occasional difficulties | 415 | 404–426 |  |  |
|  | No difficulties | 442 | 435–449 |  |  |
| Housing tenure | Renters/others | 430 | 422–437 | 0.369 | 0.544 |
|  | Homeowner | 438 | 429–446 |  |  |
| LTPA | High vigorous activity | 428 | 416–440 | 4.601 | 0.003 |
|  | Vigorous activity | 435 | 426–443 |  |  |
|  | Moderate activity | 436 | 424–448 |  |  |
|  | Low activity | 432 | 416–447 |  |  |
| Sleep sufficiency | Sufficient | 428 | 422–435 | 9.481 | 0.002 |
|  | Insufficient | 443 | 433–453 |  |  |
| Binge drinking | No binge drinking or missing | 430 | 424–436 | 6.717 | 0.010 |
|  | Weekly or more frequently | 484 | 459–508 |  |  |
| Body mass index (kg/m^2^) | <30 | 431 | 425–437 | 9.711 | 0.002 |
|  | ≥30 | 448 | 432–464 |  |  |
| Self-reported health | Good health | 430 | 424–436 | 11.009 | <0.001 |
|  | Poor health | 463 | 444–481 |  |  |
